# Examining the Effects of Installed Capacity Mix and Capacity Factor on Aggregate Carbon Intensity for Electricity Generation in China

**DOI:** 10.3390/ijerph19063471

**Published:** 2022-03-15

**Authors:** Shiping Ma, Qianqian Liu, Wenzhong Zhang

**Affiliations:** 1 Institute of Geographic Sciences and Natural Resources Research, Chinese Academy of Sciences, Beijing 100101, China; masp.17b@igsnrr.ac.cn; 2 University of Chinese Academy of Sciences, Beijing 100049, China; 3 School of Geography, Nanjing Normal University, Nanjing 210023, China; 4 Jiangsu Center for Collaborative Innovation in Geographical Information Resource Development and Application, Nanjing 210023, China

**Keywords:** electricity generation, aggregate carbon intensity, installed capacity mix, capacity factor, provincial level

## Abstract

Promoting technological advancements and energy transitions in electricity generation are crucial for achieving carbon reduction goals. Some studies have examined the effectiveness of these measures by analysing the driving forces of “aggregate carbon intensity” (ACI) change. However, only a few studies have considered the effect of the installed capacity mix and capacity factor. Moreover, such analysis has never been applied at China’s provincial level after 2015. To alleviate this gap, our study applied a temporal and multi-regional spatial IDA-LMDI model to analyse the driving factors of ACI changes and disparities among the provinces of China from 2005 to 2019. The model notably includes the effects of the installed capacity mix, thermal capacity factor, and overall capacity factor. The analysis revealed that the decline in China’s ACI was diminished after 2015, while an ACI rebound was identified in five provinces. The changes in the ACI from 2015 to 2019 were mainly driven by the effect of the installed capacity mix rather than by the thermal efficiency and thermal capacity factor. The overall capacity factor was the only factor with a negative impact on the ACI change. We also found that its combined effect with the thermal capacity factor on increasing ACI can offset the effect of the installed capacity mix by reducing the ACI in provinces with significant additions of renewable energy installed capacity. The analysis of the influencing factors on the provincial ACI differences revealed that the share of hydropower installed capacity was significant. Moreover, the thermal efficiency and thermal capacity factor both played key roles in the ACI disparities in northeast, northwest, and central China. Overall, this study paves the way for data-driven measures of China’s carbon peak and carbon neutrality goals by improving the capacity factor of wind and solar power, leveraging the critical impact of hydropower, and narrowing the differences in the thermal power sector among provinces.

## 1. Introduction

Electricity generation is the strongest contributor to carbon emissions in China given its heavy dependence on coal use. The International Energy Agency (IEA) statistics have indicated that carbon emissions from China’s electricity production sector reached 5238 MtCO_2_ in 2019, accounting for 53.0% of the national total carbon emissions [1]. It is essential to cope with the intensifying challenges of global climate change and to achieve the pledged that carbon emission will peak before 2030 by reducing carbon emissions from electricity generation [2]. China has already promoted carbon emission reduction in the electricity sector through various approaches. Technological breakthroughs in coal power, such as ultra-supercritical and advanced supercritical technologies, have significantly improved thermal efficiency, while some pilot regions have partly shifted from coal to gas in the fossil fuel mix. The development of non-fossil electricity has made some progress as well, and China stands out with the largest installed capacity and the fastest increase in renewable electricity worldwide nowadays. In particular, the installed capacities of wind power, solar power, and hydropower accounted for 10.6%, 10.0%, and 17.8% of the national total installed capacity in 2019, respectively.

The effectiveness of these transformations and the advancements in carbon emission reduction have both received considerable attention from scholars. Earlier studies directly analysed the driving forces behind the change in China’s total carbon emissions from the electricity sector at the national level or in specific provinces [3,4,5,6,7]. However, it is difficult to ignore the impact of the significant increase in electricity generation on other factors that are positively correlated with electricity generation, such as GDP and population. These studies generally indicated that economic activity or total power generation plays a vital role in increasing carbon emissions. However, the effects of technological advancements and structural transformations have not been elucidated because it is challenging to examine inter-regional differences. Recent research literature introduced an indicator, called the “aggregate carbon intensity” (ACI) of electricity generation, to measure the carbon emission performance in the power sector. ACI is defined as the CO2 emissions from fossil fuels in electricity production divided by the total electricity produced. In simple words, it is an easily quantifiable, normalised indicator that eliminates the influence of the underlying growth in electricity demand [8,9]. In some studies, the ACI has been also called “the carbon intensity of electricity generation” or “CIE” [10].

Most existing studies utilise the IDA-LMDI decomposition method to analyse the drivers of ACI changes [11]. In particular, IDA-LMDI is a conventional numerical method for analysing carbon emissions, which is easy to apply given its simple formulas, low data requirements, no residuals after decomposition, and easy cross-sectional comparison in time and spatial series [12]. The changes in ACI are usually decomposed into the thermal efficiency factor, thermal power proportion factor, fossil fuel mix factor, emission coefficient factor, and geographic distribution factor. At the country level, Ang et al. have examined the drivers of global and country-specific ACI changes from 1990 to 2013. They have reported that the marginal decrease in global ACI was driven by the effect of a geographical shift in the distribution of electricity production, whereas the thermal efficiency was the major contributor to ACI decline in most countries [8,13]. Similar studies have been conducted in the ASEAN countries from 1990 to 2014, the “Belt and Road Initiative” countries between 2013 and 2015, the Latin America and the Caribbean countries from 1990 to 2017, the United States between 2001 and 2017, and China from 1980 to 2014 [14,15,16,17,18]. The studies on China generally agreed that the decline in China’s ACI was driven by the improvement in thermal efficiency with only a minor effect of fuel change and thermal power proportion [8,17].

However, the technology and emission levels of China’s electricity sector exhibit salient regional differences, which have been often overlooked in the country-level studies [9,19]. Several studies have focused on China’s provincial disparities in ACI from electricity generation. For instance, Liu et al. have explored the driving forces of China’s provincial change in ACI from 2000 to 2014. They reported that the decline of ACI in the western regions mainly contributed to the thermal power proportion effect, while that in the eastern regions contributed to the improvement of thermal efficiency [9]. In addition to the proportion of thermal power and thermal efficiency, Wang et al. have introduced a geographic distribution factor to their analysis of a period from 1995 to 2014. They have found that the effect of this factor, as well as the thermal power proportion and thermal efficiency, on China’s provincial ACI should be taken into account [20]. Zhao et al. have innovatively introduced GDP and trade indicators into their study, suggesting that changes in provincial ACI were influenced by the effects of electricity efficiency, consumption intensity, and trade between 2001 and 2015. They also noted that the effects can be divided into four categories on the basis of the combination of these factors [19]. Notably, all of these studies have utilised the IDA-LMDI method, while underlining the complexity and variability of the driving forces of the spatio-temporal changes of China’s provincial ACI. Moreover, the decomposition results suggested that the effect of thermal efficiency had a significant impact on reducing ACI at the provincial level. Some studies have also focused only on a certain subsector. For instance, Wang et al. have utilised the Theil index in synergy with the LMDI method to analyse the ACI of the thermal power sector. They have discerned prominent intraregional and interregional variations, while its decline from 2000 to 2016 was mainly attributed to the thermal efficiency effect at the provincial level [21]. Cheng et al. have studied the influence of technical innovation in the renewable power sector on reducing ACI from 2000 to 2015 by using panel estimation methods. They reported that this impact was more pronounced in the long term than in the short term and more effective in the eastern provinces than in the western provinces [22].

As shown, three research gaps were identified. First, in the current selection of decomposition factors, the primary energy source of electricity is decomposed into fossil and non-fossil energy. This critically hampers the distinction of the respective effects of wind, solar, hydro, and other types of primary energy. Second, only a few studies have considered the installed capacity and capacity factor, which are essential indicators of electricity investment and technical improvement [23]. The thermal power proportion factor has been previously defined as the share of thermal power generation, which can directly respond to the impact of clean energy penetration on carbon reduction. However, the complex relationship between the installed capacity and capacity factor has been overlooked. As global non-fossil electricity investments are growing, the dramatic decline in capacity factor, caused by renewable energy curtailment, has become a global concern. These concerns are peculiarly acute in the countries with large wind and solar power installed capacities, such as China [24]. Due to this, it is necessary to study the impact of the installed capacity mix and capacity factors on China’s ACI. Third, only a few studies have been conducted at the regional scale in China, and only a few studies have focused on the new trend of ACI change and its driving forces since 2015.

To alleviate these gaps, our study analysed the driving forces of both the changes and disparities in China’s provincial ACI by using the IDA-LMDI decomposition method. The study was applied to the electricity sector data between 2005 and 2019 in China. Note that the effects of the installed capacity mix and capacity factor were introduced into the decomposition model alongside the conventionally used factors, such as the thermal efficiency effect and fuel switching effect. The remainder of this paper is organised as follows. Section 2 introduces the study’s methodology and data sources. Section 3 presents and analyses the decomposition results over time and space. Section 4 formulates the conclusions and policy implications for China’s carbon reduction efforts in the electricity sector.

## 2. Methodology and Data

### 2.1. Decomposition Method

In the models proposed in previous studies [23,25], the combined effect of all the drivers of the ACI of electricity generation by fuel type i in a specific region and in a selected year can be expressed as:

(1) $$V=\frac{C}{Q}=\frac{\sum_{i}C_{i}}{Q}=\sum_{i}\frac{C_{i}}{F_{i}}\times \frac{F_{i}}{F}\times \frac{F}{Q_{f}}\times \frac{Q_{f}}{8760K_{f}}\times \frac{K_{f}}{K}\times \frac{8760K}{Q}$$

where V is the ACI from electricity generation, C is the total CO_2_ emissions from all fossil fuel types, and $C_{i}$ is the CO_2_ emissions from fossil fuel i; $F_{i}$ is the energy input from fossil fuel i; F is the energy input from all types of fossil fuels, Q is the total electricity generation, $Q_{f}$ is the thermal power generation, K is the total installed capacity, and $K_{f}$ is the installed capacity of fossil-based plants. According to the setting of the model, the carbon emissions of power production are only generated by fossil fuel combustion in the thermal power sector. The power production process from other sources does not emit carbon dioxide, but will change the ACI of the whole electricity sector.

The decomposition factors are defined as follows:

$e_{i}=\frac{C_{i}}{F_{i}}$ is the emission coefficient factor for fossil fuel i. According to the IPCC method, this factor is calculated according to the calorific value, carbon content, and the fraction of carbon oxidised, and such data were collected from the *China Energy Statistical Yearbook* [26] and the *Guidelines for Provincial Greenhouse Gas Inventories (Pilot Version)* [27]. All indicators were converted to standard coal equivalents and remained constant across space and periods, as shown in Table 1.

$e_{i}$$f=\frac{F}{Q_{f}}$ is thermal efficiency, which is defined as the ratio of fossil fuel input to thermal electricity generation. $q_{i}=\frac{F_{i}}{F}$ is the fossil fuel mix, defined as the share of energy input from fossil fuel i in the total energy input; $k_{f}=\frac{Q_{f}}{8760K_{f}}$ is the thermal capacity factor, calculated by dividing the actual thermal electricity generation by the maximum possible output of the thermal plants; $k=\frac{Q}{8760K}$ is the overall capacity factor, calculated by dividing the total electricity generation by the maximum possible output of all types of plants; $p=\frac{K_{f}}{K}$ is the installed capacity mix, defined as the share of the installed thermal capacity against the total installed capacity.

To further distinguish the effect of the installed capacity mix on ACI, the proportion of the thermal installed capacity can also be decomposed into four terms according to Wang et al. [28]:

(2) $$\begin{array}{ll} p & =\frac{K_{f}}{K}=\frac{K_{f}}{K_{f}+K_{s}}\times \frac{K_{f}+K_{s}}{K_{f}+K_{s}+K_{w}}\times \frac{K_{f}+K_{s}+K_{w}}{K_{f}+K_{s}+K_{w}+K_{h}}\times \frac{K_{f}+K_{s}+K_{w}+K_{h}}{K}\\ & =s\times w\times h\times n \end{array}$$

where $K_{s}$, $K_{w}$, and $K_{h}$ denote the installed capacities of the solar power, wind power, and hydropower, respectively; s, w, h, and n stand for the effect of installed capacity from solar power, wind power, hydropower, and others, respectively. Thus, Equation (1) can be rewritten as a function of the nine effects:

(3) $$V=\sum_{i}e_{i}\times f\times q_{i}\times k_{f}\times s\times w\times h\times n\times \frac{1}{k}$$

Based on the additive IDA-LMDI approach [29], the changes in ACI in the research areas from year t−1 to year t can be decomposed as:

(4) $$\begin{array}{ll} V & =V^{t}-V^{t-1}=\Delta V_{e}+\Delta V_{f}+\Delta V_{q}+\Delta V_{k_{f}}+\Delta V_{p}+\Delta V_{k}\\ & =\Delta V_{e}+\Delta V_{f}+\Delta V_{q}+\Delta V_{k_{f}}+\Delta V_{s}+\Delta V_{w}+\Delta V_{h}+\Delta V_{n}+\Delta V_{k} \end{array}$$

where $\Delta V_{e}$, $\Delta V_{f}$, $\Delta V_{q}$, $\Delta V_{k_{f}}$, $\Delta V_{p}$, $\Delta V_{k}$, $\Delta V_{s}$, $\Delta V_{w}$, $\Delta V_{h}$, and $\Delta V_{n}$ are the impacts associated with variations in e, f, q, $k_{f}$, p, k, s, w, h, and n from year t−1 to year t. The decomposition formulae for these impacts are as follows. $\Delta V_{e}$ is omitted because it remains constant at 0.

(5) $$\begin{array}{l} \Delta V_{f}=\sum_{i}\frac{V_{i}^{t}-V_{i}^{t-1}}{\ln(V_{i}^{t})-\ln(V_{i}^{t-1})}\times \ln(\frac{f^{t}}{f^{t-1}})\\ \Delta V_{q}=\sum_{i}\frac{V_{i}^{t}-V_{i}^{t-1}}{\ln(V_{i}^{t})-\ln(V_{i}^{t-1})}\times \ln(\frac{q_{i}^{t}}{q_{i}^{t-1}})\\ \Delta V_{k_{f}}=\sum_{i}\frac{V_{i}^{t}-V_{i}^{t-1}}{\ln(V_{i}^{t})-\ln(V_{i}^{t-1})}\times \ln(\frac{k_{f}^{t}}{k_{f}^{t-1}})\\ \Delta V_{p}=\sum_{i}\frac{V_{i}^{t}-V_{i}^{t-1}}{\ln(V_{i}^{t})-\ln(V_{i}^{t-1})}\times \ln(\frac{p_{}^{t}}{p_{}^{t-1}})\\ \Delta V_{k}=\sum_{i}\frac{V_{i}^{t}-V_{i}^{t-1}}{\ln(V_{i}^{t})-\ln(V_{i}^{t-1})}\times \ln(\frac{k_{}^{t-1}}{k_{}^{t}})\\ \Delta V_{s}=\sum_{i}\frac{V_{i}^{t}-V_{i}^{t-1}}{\ln(V_{i}^{t})-\ln(V_{i}^{t-1})}\times \ln(\frac{s_{}^{t}}{s_{}^{t-1}})\\ \Delta V_{W}=\sum_{i}\frac{V_{i}^{t}-V_{i}^{t-1}}{\ln(V_{i}^{t})-\ln(V_{i}^{t-1})}\times \ln(\frac{w_{}^{t}}{w_{}^{t-1}})\\ \Delta V_{h}=\sum_{i}\frac{V_{i}^{t}-V_{i}^{t-1}}{\ln(V_{i}^{t})-\ln(V_{i}^{t-1})}\times \ln(\frac{h_{}^{t}}{h_{}^{t-1}})\\ \Delta V_{n}=\sum_{i}\frac{V_{i}^{t}-V_{i}^{t-1}}{\ln(V_{i}^{t})-\ln(V_{i}^{t-1})}\times \ln(\frac{n_{}^{t}}{n_{}^{t-1}}) \end{array}$$

To explore the contributors to the differences in ACI across China’s provinces, we also decomposed the discrepancies in ACI between province j and national average 0 in 2019. To this end, we utilised the multi-regional spatial decomposition model from [30]:

(6) $$\begin{array}{ll} V & =V^{j}-V^{0}=\Delta V_{e}^{'}+\Delta V_{f}^{'}+\Delta V_{q}^{'}+\Delta V_{k_{f}}^{'}+\Delta V_{p}^{'}+\Delta V_{k}^{'}\\ & =\Delta V_{e}^{'}+\Delta V_{f}^{'}+\Delta V_{q}^{'}+\Delta V_{k_{f}}^{'}+\Delta V_{s}^{'}+\Delta V_{w}^{'}+\Delta V_{h}^{'}+\Delta V_{n}^{'}+\Delta V_{k}^{'} \end{array}$$

where $\Delta V_{e}^{'}$, $\Delta V_{f}^{'}$, $\Delta V_{q}^{'}$, $\Delta V_{k_{f}}^{'}$, $\Delta V_{p}^{'}$, $\Delta V_{k}^{'}$, $\Delta V_{s}^{'}$, $\Delta V_{w}^{'}$, $\Delta V_{h}^{'}$, and $\Delta V_{n}^{'}$ are the impacts associated with discrepancies of e, f, q, $k_{f}$, p, k, s, w, h, and n between province j and the national average 0. The decomposition formulae for these impacts are shown in Equation (7), where $\Delta V_{e}^{'}$ was omitted because it remained constant at 0.

(7) $$\begin{array}{l} \Delta V_{f}^{'}=\sum_{i}\frac{V_{i}^{j}-V_{i}^{0}}{\ln(V_{i}^{j})-\ln(V_{i}^{0})}\times \ln(\frac{f^{j}}{f^{0}})\\ \Delta V_{q}^{i}=\sum_{i}\frac{V_{i}^{j}-V_{i}^{0}}{\ln(V_{i}^{j})-\ln(V_{i}^{0})}\times \ln(\frac{q_{i}^{j}}{q_{i}^{0}})\\ \Delta V_{k_{f}}^{'}=\sum_{i}\frac{V_{i}^{j}-V_{i}^{0}}{\ln(V_{i}^{j})-\ln(V_{i}^{0})}\times \ln(\frac{k_{f}^{j}}{k_{f}^{0}})\\ \Delta V_{p}^{'}=\sum_{i}\frac{V_{i}^{j}-V_{i}^{0}}{\ln(V_{i}^{j})-\ln(V_{i}^{0})}\times \ln(\frac{k_{}^{0}}{k_{}^{j}})\\ \Delta V_{k}^{'}=\sum_{i}\frac{V_{i}^{j}-V_{i}^{0}}{\ln(V_{i}^{j})-\ln(V_{i}^{0})}\times \ln(\frac{k_{}^{0}}{k_{}^{j}})\\ \Delta V_{s}^{'}=\sum_{i}\frac{V_{i}^{j}-V_{i}^{0}}{\ln(V_{i}^{j})-\ln(V_{i}^{0})}\times \ln(\frac{s_{}^{j}}{s_{}^{0}})\\ \Delta V_{w}^{'}=\sum_{i}\frac{V_{i}^{j}-V_{i}^{0}}{\ln(V_{i}^{j})-\ln(V_{i}^{0})}\times \ln(\frac{w_{}^{j}}{w_{}^{0}})\\ \Delta V_{h}^{'}=\sum_{i}\frac{V_{i}^{j}-V_{i}^{0}}{\ln(V_{i}^{j})-\ln(V_{i}^{0})}\times \ln(\frac{h_{}^{j}}{h_{}^{0}})\\ \Delta V_{n}^{'}=\sum_{i}\frac{V_{i}^{j}-V_{i}^{0}}{\ln(V_{i}^{j})-\ln(V_{i}^{0})}\times \ln(\frac{n_{}^{j}}{n_{}^{0}}) \end{array}$$

### 2.2. Data Sources

We used data on the installed capacity of power delineated into thermal power, solar power, wind power, hydropower, and power from other sources. The data on the electricity generation of thermal power and non-fossil power alongside the energy consumption of three types of fossil fuels were also utilised. Both types of data covered China’s 30 mainland provinces, without Tibet because of the lack of data for 2005, 2010, 2015, and 2019. The installed capacity and electricity generation data were collected from the *China Electricity Statistical Yearbook* [26] and the *Statistical Compilation of the China Electricity Industry* [31]. Energy consumption data were collected from the *China Energy Statistical Yearbook* [26]. The zero values were replaced by $\delta =10^{-100}$ under the “small value” (SV) strategy, according to the experience of previous studies [32,33].

We analysed the contributors to the changes in provincial ACI from 2005 to 2010, 2010 to 2015, and 2015 to 2019. These intervals were consistent with the schedules for China’s Five-Year Plans. (The Five-Year Plan (FYP) is a strategic plan for economic, social, and environmental development in China. The 11th FYP covered the years 2005 to 2010. The 12th FYP spanned from 2010 to 2015, and the 13th FYP covered the years 2015 to 2020.) In this way, the temporal resolution of the study is aligned with the policy-relevant periods. Note that the supply and demand market for fossil fuels significantly changed circa 2010 and the investment in renewable power entered a period of the rapid growth. As 2015 was the last year covered by the previous studies, we extended the study period from 2015 to 2019. To provide valuable region-specific policy implications, we also studied the driving forces behind the differences in provincial ACI in 2019.

## 3. Results and Analysis

### 3.1. Overview of ACI Changes

We found that China’s ACI of electricity generation reached 571.87 g·CO_2_/kWh in 2019. The decline in the national ACI from 2015 to 2019 became significantly smaller at 39.62 g·CO_2_/kWh, while the declines in the previous two time periods both exceeded 100 g·CO_2_/kWh. At the provincial level, the ACI of each province decreased continuously during the first two periods, but the vector of change was not consistent in the last period. More specifically, Guangxi, Guizhou, Hubei, Shanxi, and Chongqing experienced a rebound in ACI from 2015 to 2019, with the highest increase of 155.21 g·CO_2_/kWh in Guangxi. The ACI in Hunan and Inner Mongolia remained largely unchanged between 2015 and 2019, with a decreases of <2.00 g·CO_2_/kWh. The statistical analysis revealed that China’s provinces exhibited wide disparities in the ACI of electricity generation, as shown in Table 2. The inter-provincial differences have somewhat increased since 2010, with more palpable variability (e.g., higher standard deviations). They reached 860.06 g·CO_2_/kWh in 2015 and 851.54 g·CO_2_/kWh in 2019.

**Figure 1:**
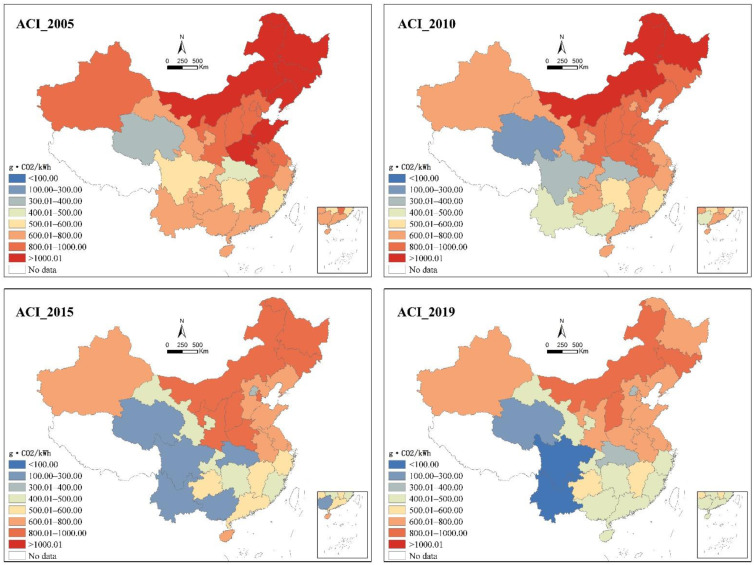
The spatial distribution of provincial ACI in China.

The spatial analysis of the provincial ACI in China revealed a pattern of high values in north and northeast China, while low values were identified in northwest, southwest, and south China from 2010 (Figure 1). In 2019, Inner Mongolia stood out with the highest ACI, reaching 936.00 g·CO_2_/kWh, followed by Jilin and Shanxi (ACI > 800 g·CO_2_/kWh). Meanwhile, the ACI of Yunnan, Sichuan, and Qinghai was found to be much lower than other provinces, with ACI values of 84.46 g·CO_2_/kWh, 86.13 g·CO_2_/kWh, and 101.22 g·CO_2_/kWh, respectively. Beijing exhibited the lowest ACI (317.56 g·CO_2_/kWh) among the northern provinces, being ranked fourth in the ascending order among all the analysed provinces. Of the four provinces with the lowest level of aggregate carbon intensity, Qinghai exhibited a steadily low ACI since 2005. Meanwhile, the other three provinces had experienced dramatic changes, with a decline in ACI between 2005 and 2019 of 86.36%, 84.94%, and 61.95% in Yunnan, Sichuan, and Beijing, respectively.

### 3.2. Temporal Decomposition Results

**Figure 2:**
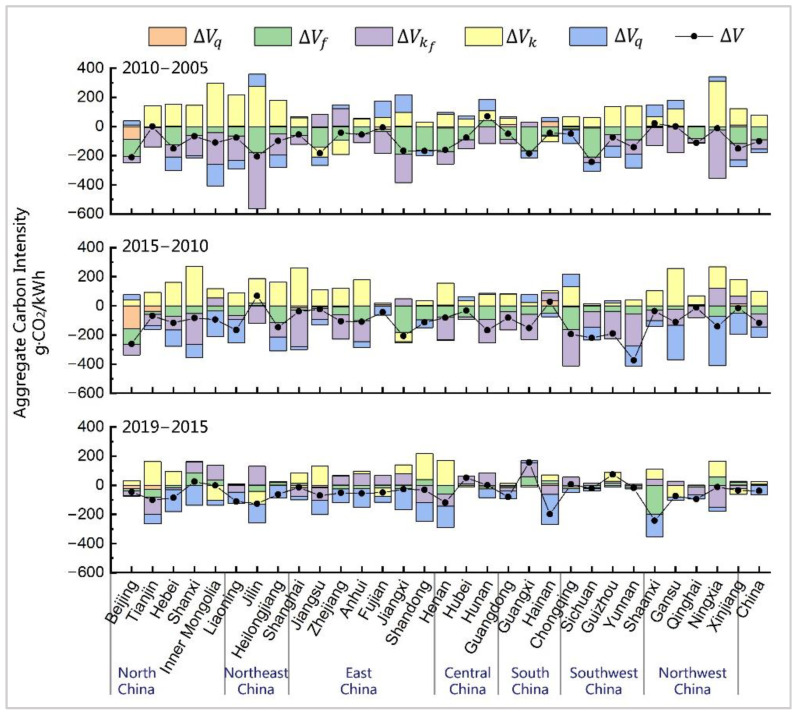
Performance of driving factors in provincial temporal IDA-LMDI.

The temporal decomposition (Figure 2) revealed that the effects of the studied factors on the ACI changes greatly varied in time and space. For clarity and objectiveness, we divided all the effects into three categories: (1) the traditional effects, including thermal efficiency ($\Delta V_{f}$) and fossil fuel mix ($\Delta V_{q}$), which also appeared in existing studies before 2015; (2) the structural shift effects, consisting of the installed capacity mix ($\Delta V_{p}$) alongside the respective effect of non-fossil power from various primary energy sources ($\Delta V_{s}$, $\Delta V_{w}$, $\Delta V_{h}$, and $\Delta V_{n}$), and (3) the capacity factor effects, which includes the thermal capacity factor ($\Delta V_{k_{f}}$), overall capacity factor ($\Delta V_{k}$), and their combined effect.

#### 3.2.1. Traditional Effects

The decline in the thermal efficiency ($\Delta V_{f}$) was an important contributor to the substantial drop in national and provincial ACI before 2015 (Figure 2). This finding is in line with the results from previous studies [19,34]. China’s thermal efficiency decreased from 378.99 gce/kWh in 2005 to 312.63 gce/kWh in 2015 under the joint efforts of each province, thereby causing the overall decrease of >20% in each province, with a decrease of 65.66% in total. The significant improvement in thermal efficiency was mainly driven by the technological breakthroughs, such as ultra-supercritical and advanced supercritical technology, and shutting down small old thermal power units during this period. However, the thermal efficiency factor was no longer the main driving force for the ACI changes in most provinces in the 2015–2019 period ($\Delta V_{f}^{19-15}$ = 1.48 g·CO_2_/kWh). This was merely attributable to the ACI drop in Shaanxi, given its significant decline in thermal efficiency of 23.31%. Unlike the previous two periods, 12 provinces experienced a thermal efficiency rebound during this period, with the largest increases of 46.84 gce/kWh in Guangxi, 35.71 gce/kWh in Shanxi, and 24.54 gce/kWh in Ningxia, which elevated the local ACI up to 59.64 g·CO_2_/kWh, 86.48 g·CO_2_/kWh, and 55.49 g·CO_2_/kWh, respectively.

The analysis of the fossil fuel mix factor ($\Delta V_{q}$) at the national scale revealed that its effect was very weak throughout the entire period. Meanwhile, at the provincial scale, it was only effective in Beijing before 2015 (Figure 2). Specifically, Beijing experienced a radical shift in the fossil fuel mix during this period, with an increasing share of natural gas consumption from 0.51% to 85.41%. This corresponded to the reduction of the ACI by 245.69 g·CO_2_/kWh, thereby accounting for 52.12% of the total decrease from 2005 to 2015. The impact of the fossil fuel mix on China’s ACI reduction has received little attention in previous studies, but its effectiveness in Beijing was proven, as it has become one of the cleanest provinces from an electricity generation perspective.

#### 3.2.2. Structural Shift Effects

The decline in the proportion of thermal installed capacity in the total installed capacity ($\Delta V_{p}$) became a critical driving factor for ACI drop in the provinces of north, northeast, and northwest China between 2010 and 2015. During the third period, the national ACI decrease was mainly driven by this factor ($\Delta V_{p}^{19-15}$ = −63.60 g·CO_2_/kWh). Moreover, we found that it was the major contributor to the ACI decrease in most provinces (see Figure 2).

**Figure 3:**
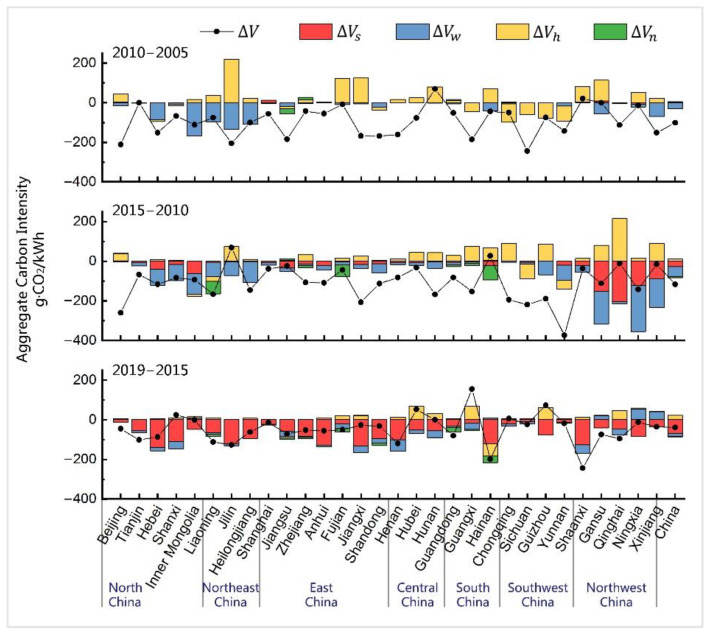
Performance of respective effects of different kinds of non-fossil power.

Figure 3 illustrates the respective impacts of power from different primary energy sources on the installed capacity mix. The increase in the wind power installed capacity share ($\Delta V_{w}$) had a positive impact on the ACI reduction in north, northeast, and northwest China from 2005 to 2015, whereas the provinces in northwest China were also affected by solar power ($\Delta V_{s}$) during the same period. From 2015 to 2019, the impacts on the decline in ACI were mainly driven by the substantial supplementation of the solar power installed capacity (e.g., from 42.02 million kW to 203.10 million kW in total). However, the effect of wind power became relatively small. Furthermore, the spatial distribution of solar power installation was not as clustered as that of wind power, with a common positive effect on the ACI reduction in all the provinces of China. From the hydropower sector ($\Delta V_{h}$) perspective, the share of hydropower installed capacity continued decreasing throughout the first two periods in most provinces, excluding those in southwest China. As the hydropower development levels in areas excluding southwest China have reached about 50%, the technical difficulty and environmental cost of the remaining exploitable capacity are growing, which leads to the increase in the hydropower construction cost. At the same time, the high-level subsidy policies for wind power and solar power in recent years also reduce the willingness for hydropower development in these areas. [35,36]. The decline in the proportion of hydropower installed capacity caused a moderate increase in the ACI in most provinces before 2015. However, its slight impact completely disappeared in the last five years. Compared to the abovementioned types of renewable power, the influence of nuclear power and other sources of power ($\Delta V_{n}$) was somewhat weak at both the national and provincial scales. Given the considerations of elements such as construction costs and safety, only eight provinces have nuclear power units nowadays. Their limited and nearly unchanged installed capacity had only a little impact on the ACI change.

#### 3.2.3. Capacity Factor Effects

**Figure 4:**
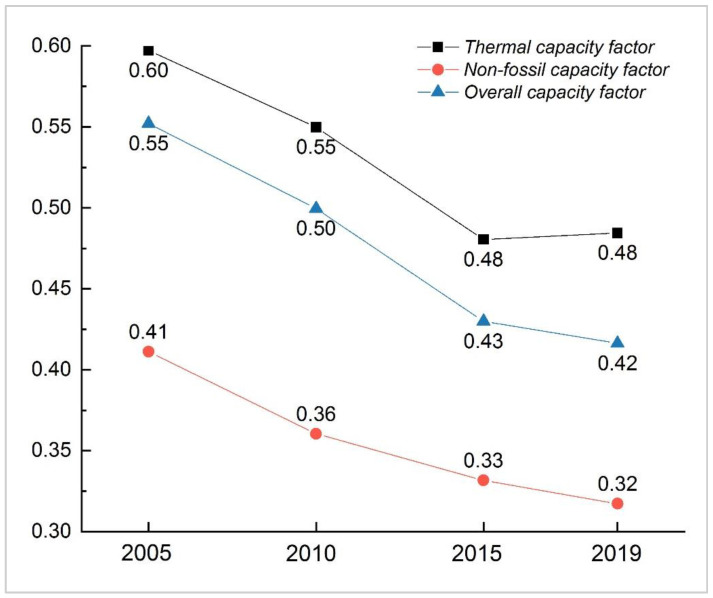
The change in China’s capacity factors.

The thermal capacity factor ($\Delta V_{k_{f}}$) was another critical driver behind the decline in ACI before 2015. China’s thermal capacity factor plummeted from 0.60 to 0.48 during the first two periods (Figure 4), thereby decreasing the national ACI by 154.07 g·CO_2_/kWh in total. However, 16 provinces exhibited a thermal capacity factor rebound from 2015 to 2019, with the largest growth of 0.12 in Guangxi, which increased the provincial ACI by 94.62 g·CO_2_/kWh. Similar to the thermal efficiency effect, the thermal capacity factor not only contributed to the ACI reduction, but also slightly exacerbated its growth from 2015 to 2019. The impact of the thermal power sector on carbon emission reductions was at a standstill after 2015.

The overall capacity factor ($\Delta V_{k}$) was the only factor that affected the ACI change in a direction opposite to its own change. This indicates that the low level of overall utilisation of power units was not conducive to reducing carbon emissions. At the national level, the decrease in the overall capacity factor significantly drove the increase in China’s ACI during the first two periods. As shown in Figure 4, the overall capacity factor dropped from 0.55 to 0.43 from 2005 to 2015 due to the decrease in the capacity factors of both the thermal and non-fossil power sectors. This decline induced the increase in the ACI by 177.80 g·CO_2_/kWh in total. From 2015 to 2019, the impact of the overall capacity factor on national ACI became small, which was merely attributable to the decline of the non-fossil capacity factor from 0.33 to 0.32. However, this impact was still manifested as the major driver of the national ACI increase. From a spatial perspective (see Figure 2), the negative impact of the overall capacity factor on the ACI deduction gradually expanded from the northern provinces to east and central China during the previous two periods. From 2015 to 2019, the influence of the overall capacity factor started weakening, and its change vectors in a few provinces were no longer consistent with those of previous periods. We also identified an increase in the overall capacity factor in Inner Mongolia, Jilin, Fujian, Gansu, and Xinjiang, which caused a somewhat significant decrease in their ACI.

**Figure 5:**
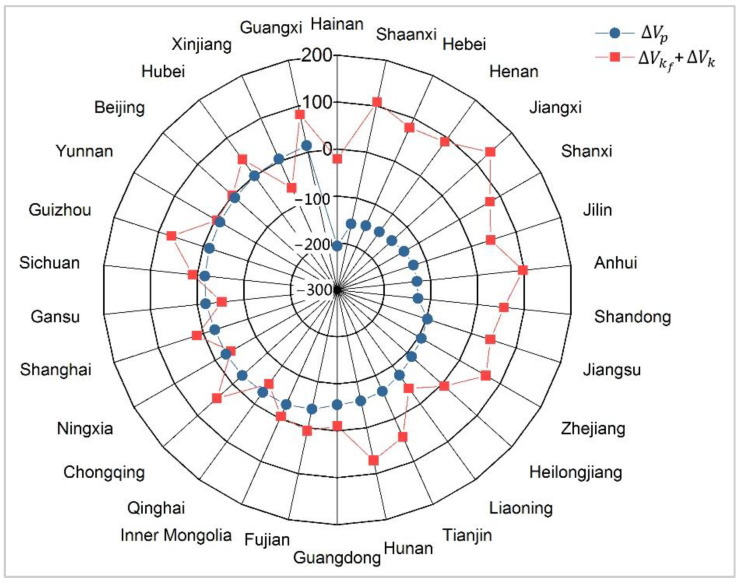
Relationship between the effect of installed capacity mix and capacity factors from 2015 to 2019.

As the decline in the thermal capacity factor can induce a decrease in the overall capacity factor, and they have an inverse impact on the ACI changes, we combined the effects of the overall capacity factor and the thermal capacity factor ($\Delta V_{k}$ + $\Delta V_{k_{f}}$). According to the decomposition equation in Section 2.1, the combined effect represents the impact of the non-fossil capacity factor. As shown in Figure 5, the effect of the non-fossil capacity factor ($\Delta V_{k}$ + $\Delta V_{k_{f}}$) on increasing the ACI was found to be more prominent in the provinces where the installed capacity mix significantly impacted the ACI decline in 2015–2019. In other words, the rapid growth of renewable power installed capacity may have great potential for reducing ACI, but the associated decrease in the capacity factor would inevitably offset its effect on ACI reduction.

We further analysed the characteristics of a structural shift in the installed capacity mix of the 11 provinces with the largest installed capacity mix effect (Table 3). Besides the Hainan province, the other ten provinces exhibited a rapid increase in solar power installed capacity, with a share of 60.97% of the total national addition in solar power capacity. The addition of Shandong alone has led to a 9.23% increase at the national level, while Jilin and Henan exhibited 39- and 26-fold increases, respectively. Shaanxi, Henan, Shanxi, Shandong, and Jiangsu also exhibited rapid growth in wind power installed capacity. Of these provinces, Henan exhibited the fastest growth rate, with its installed capacity increasing by 8.73 times, as well as the largest increase, accounting for 9.0% of the total national addition of wind power installed capacity. By contrast, the amount and rate of hydropower growth in these provinces were not significant. It can be inferred that the offset effect of the non-fossil capacity factor on reducing ACI was mainly driven by the prevalence of solar power in the installed capacity supplements of non-fossil power. Notably, it exhibited the lowest average capacity factor (0.12), compared to that of wind power (0.22), hydropower (0.31), and thermal power (0.41). This finding can lay the foundation for policy implications, as increasing renewable power installations and improving the capacity factor can lead to the best emission reduction effect only if they are implemented together.

### 3.3. Spatial Decomposition Results

**Figure 6:**
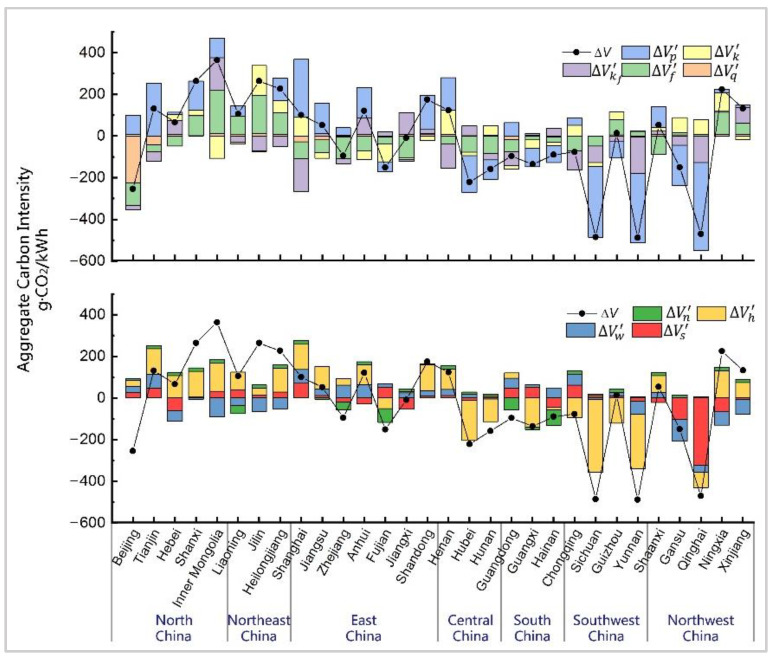
Performance of driving factors in spatial IDA-LMDI decomposition.

**Figure 7:**
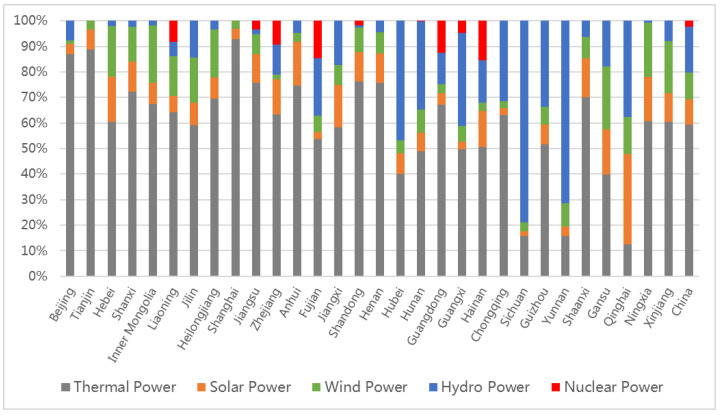
Share of power installed capacity from different primary energies in 2019.

The multi-regional spatial decomposition model, shown in Section 2.1, was applied to provide an insight into the driving forces behind the discrepancy in the provincial ACI in 2019. Figure 6 shows that the differences in the installed capacity mix significantly affected the discrepancy in the provincial ACI. The analysis of the primary energy source revealed that the share of hydropower installed capacity was the major contributor to the disparities in the ACI. As shown in Figure 7, the proportion of hydropower installed capacity in each province significantly differed. The share of hydropower installed capacity in 2019 was 17.76% at the national level, while that in the provinces of central, south, and southwest China was significantly higher. Sichuan exhibited the highest proportion of hydropower installed capacity at 79.02%, followed by 71.44% in Yunnan and 46.79% in Hubei, thereby reducing the ACI by 349.01 g·CO_2_/kWh, 264.70 g·CO_2_/kWh, and 194.46 g·CO_2_/kWh, respectively. Although the previous analysis proved that the impact of hydropower on the recent ACI reduction was not as strong as that of other renewable power sources, its impact on the identified provincial differences was great. In contrast, solar power only contributed to the low ACI in Qinghai and Gansu, while wind power had little impact on the ACI differences between the provinces.

Thermal efficiency and the thermal capacity factor also represented the important factors, contributing to the provincial ACI differences, despite Section 3.2 demonstrating that they had a negligible impact on ACI changes over the last five years. The thermal efficiencies of twelve provinces were higher than the national average, seven of which exceeded the national average by more than 10%: Inner Mongolia (+33.00%), Jilin (+30.66%), Ningxia (+17.16%), Heilongjiang (+16.83%), Liaoning (+15.21%), Shanxi (+14.61%), and Guizhou (+13.50%). In Inner Mongolia, the impact of thermal efficiency has pulled the ACI up by 209.17 g·CO_2_/kWh relative to the national average. In Jilin, this impact reached 184.80 g·CO_2_/kWh. The thermal capacity factor analysis demonstrated that there were thirteen provinces with a thermal capacity factor higher than that of the national average, six of which exceeded the national average by more than 10%: Inner Mongolia (+23.32%), Jiangxi (20.55%), Anhui (+13.40%), Xinjiang (+12.57%), Hebei (+11.56%), and Hubei (+10.84%). In Inner Mongolia, the thermal capacity factor induced an ACI increase of 153.74 g·CO_2_/kWh. By contrast, in the provinces with a lower thermal capacity factor, such as Shanghai and Yunnan, this factor decreased the ACI by 158.16 g·CO_2_/kWh and 176.06 g·CO_2_/kWh, respectively. Compared to the driving forces mentioned above, the effect of the overall capacity factor on the provincial ACI difference was relatively small, and the impact of the fossil fuel mix was somewhat limited in Beijing.

## 4. Policy Recommendations

The findings of our study can lay the foundations for the following policy recommendations that can lead to the reduction of carbon emissions at the national and provincial levels in China.

(1) Develop strategies to improve the capacity factor of wind and solar power alongside investment and construction. To achieve the commitment that carbon dioxide emissions peak before 2030, the Chinese central government has set a target, on the basis of which the installed capacity of wind and solar power must reach 1.2 billion kW by 2030 [37]. In theory, the most effective way to reduce carbon emissions is to shift from thermal to renewable power. However, international experience indicates that the problem of curtailment would be further exacerbated by the expansion of renewable power installed capacity [38]. Several studies have pointed out that China faces severe wind and solar energy curtailment due to natural factors, such as resource abundance and atmospheric conditions, and human-related factors, such as technology and institutions [39,40]. Specifically, China surpassed the United States in 2010 to become the nation with the world’s largest installed wind power capacity, but could not surpass the United States in wind power generation by 2016 [41]. From a solar power perspective, China surpassed Germany in 2015 to be the country with the world’s largest installed solar power capacity and generation, but the capacity factor of solar power units remained below that of the United States, Japan, and India [42]. Future supplements to the renewable power installed capacity may further weaken the overall capacity factor and may critically hamper the full positive impact on ACI reduction. Therefore, it is necessary to improve the capacity factor of renewable power through tailored planning, increasing the efficiency of local power consumption, and constructing cross-regional power transmission channels and other effective initiatives [43].

(2) The critical impact of hydropower on reducing ACI needs to be further emphasised and leveraged. Some studies have reported that, although China had experienced large-scale hydropower capacity construction, the hydropower development rate of theoretical reserves is considerably lower than that in Europe and the United States. For example, the Yangtze River basin, with the largest hydropower installed capacity in China, has a development level of 25.6% of its technical exploitation amount [36]. Thus, there is room for future investment and construction. More specifically, the current hydropower development has encountered some difficulties, such as the negative impact on the environment, encroachment on investment from wind and solar power, and hydropower curtailment [35]. The dominant effect of hydropower on provincial ACI differences has been confirmed by our analysis, despite the fact that its share has been annually decreasing. In the future, more efforts are required to further promote hydropower construction, coordinate the development of hydropower with other renewable power sources, and strengthen the hydropower capacity factor; all these steps will help to reduce the ACI and carbon emissions more effectively.

(3) Finally, we must narrow the differences in the thermal power sector among provinces and avoid further rebound in the ACI from thermal power generation. Our analysis revealed that changes in the thermal power sector significantly affected the ACI reduction, but the improvement in thermal power stagnated from 2015 to 2019. Therefore, more effective means are currently needed to stimulate technological advancements in the thermal power sector. China’s National Development and Reform Commission recently announced that national thermal efficiency should be controlled below 300 gce/kWh by 2025 [44]. A total of 13 provinces exhibited a thermal efficiency of >300 gce/kWh in 2019, whereas most provinces were located in northern and western China. On the one hand, local governments, enterprises, and research institutions should be involved in the cutting-edge research on thermal power technology. On the other hand, technologies transference from the eastern provinces to the northern and western provinces must be accelerated through tailored support to narrow the regional disparities. Strict control of technical standards for new thermal power units is also needed to prevent rebounds in thermal efficiency. Moreover, advancing the flexible transformation can help in coordinating the thermal power and new energy power and can aid in controlling the thermal capacity factor. The provinces with sufficient natural gas supply should moderately develop natural gas power to weaken the share of coal in fossil fuel consumption. Namely, they can apply the experience and knowledge gained from ACI reduction in Beijing to increase the efficiency of the provincial carbon abatement activities.

## 5. Conclusions

China has already invested much effort into reducing carbon emissions from electricity generation, which necessitates the formulation of tailored policy instruments for abating carbon. This study utilised the concept of “Aggregate Carbon Intensity” (ACI) to quantify the carbon emission level of electricity generation. We quantified the ACI in China at national and provincial level for the 2005–2019 period. From a methodological perspective, the IDA-LMDI method was applied to elucidate the drivers of the ACI changes in each five-year interval and provincial differences in 2019. Compared with previous studies, we introduced new decomposition factors into the analysis, such as the effect of the installed capacity mix, the thermal capacity factor, and the overall capacity factor, and we updated the study period to 2019.

We found that the decline in ACI experienced in 2015–2019 was significantly smaller than that before, while an ACI rebound was identified in five provinces. The spatial distribution of the provincial ACI was high in north and northeast China and low in northwest, southwest, and south China in 2019, with broader inter-provincial disparities. The decomposition results are listed as follows: First, since 2010, the effect of the installed capacity mix has become the main contributor to both ACI changes and provincial differences. The analysis of primary energy sources revealed that the rapid national-scale supplements to the solar power installed capacity all over China caused the reduction in the provincial ACI after 2015. In contrast, the impact of wind power was limited to the northern provinces and further declined over the last five years. However, the share of hydropower installed capacity (not solar or wind power) governed the ACI differences between provinces. Second, the decline in the overall capacity factor had a prominent negative impact on the ACI reductions before 2015. Notably, the directions of change were no longer consistent in 2015–2019. Furthermore, the combined effect of the overall capacity factor and thermal capacity factor exhibited an offset impact on the provincial ACI reduction, given the rapid growth of the non-fossil power installed capacity, especially of solar power. Third, the effect of thermal efficiency and thermal capacity factor lost its dominant role in the ACI reduction after 2015, but exhibited a rebound that promoted the increase in ACI. The thermal efficiency rebound occurred in 12 provinces, whereas the thermal capacity factor rebound was more common and occurred in 16 provinces. Nevertheless, these two factors were found to be the main drivers behind the provincial ACI differences in northeast, northwest, and central China.

The current research only decomposed and analysed the contribution of various influencing factors on the basis of historical data. In the future, it is still necessary to deeply analyse the mechanism behind these mathematical relationships and predict the change in ACI in the future. In addition, limited by the availability of research data, this study does not focus on the biomass power sector. Biomass power is regarded as one of the critical sectors for carbon reduction in electricity production, which should be considered in further research.

## Figures and Tables

**Table 1:** Emission Coefficient Factor.

	Raw Coal	Oil	Natural Gas
Average calorific value (kjoule/kg, kj/cu.m)	20,908	41,816	35,585
Carbon content (t·C/TJ)	26.37	20.08	15.32
Fraction of carbon oxidised	95%	98%	99%
Conversion factor (kgce/kg, kgce/cu.m)	0.71	1.43	1.22
$e_{i}$ (106 t·CO_2_/tce)	2.69	2.11	1.63

**Table 2:** Statistical information of provincial ACI.

V(g·CO_2_/kWh)	2005	2010	2015	2019
Max	1141.24	1030.87	962.72	936.00
Min	319.37	207.41	102.66	84.46
Range	821.97	823.46	860.06	851.54
S.D.	219.06	213.22	242.10	221.49

**Table 3:** Ratio and rate of non-fossil installed capacity growth in selected provinces.

j	$\frac{K_{s\_19}^{j}}{\sum_{j}K_{s\_19}^{j}}$	$\frac{K_{w\_15}^{j}}{\sum_{j}K_{w\_15}^{j}}$	$\frac{\Delta K_{s}^{j}}{\sum_{j}\Delta K_{s}^{j}}.\;$	$\frac{K_{s\_19}^{j}}{K_{s\_15}^{j}}.\;$	$\frac{\Delta K_{w}^{j}}{\sum_{j}\Delta K_{w}^{j}}.\;$	$\frac{K_{w\_19}^{j}}{K_{w\_15}^{j}}.\;$	$\frac{\Delta K_{h}^{j}}{\sum_{j}\Delta K_{h}^{j}}.\;$	$\frac{K_{h\_19}^{j}}{K_{h\_15}^{j}}$
Hainan	0.64%	0.14%	0.70%	8.06	−0.03%	0.94	2.41%	2.48
Shaanxi	4.62%	2.54%	5.38%	13.04	5.33%	4.67	3.28%	1.47
Hebei	7.26%	7.84%	7.77%	6.64	7.87%	1.60	0.00%	1.00
Henan	5.19%	3.80%	6.29%	25.71	8.96%	8.73	0.24%	1.02
Jiangxi	3.10%	1.37%	3.64%	14.65	2.79%	4.27	4.48%	1.35
Shanxi	5.36%	5.98%	6.07%	9.80	7.42%	1.87	−0.55%	0.91
Jilin	1.35%	2.66%	1.66%	39.14	1.44%	1.25	1.78%	1.18
Anhui	6.17%	1.31%	7.03%	10.36	1.76%	2.01	1.42%	1.19
Shandong	7.97%	6.47%	9.23%	12.17	8.07%	1.88	0.00%	1.00
Jiangsu	7.32%	4.98%	6.61%	3.52	8.02%	2.53	3.96%	2.32
Zhejiang	6.59%	0.76%	7.29%	8.16	0.71%	1.54	4.40%	1.17
China	100.00%	100.00%	100.00%	4.83	100.00%	1.60	100.00%	1.12

## Data Availability

Publicly available datasets were analyzed in this study. This data can be found here: https://data.cnki.net/.
